# Graphene quantum interference photodetector

**DOI:** 10.3762/bjnano.6.74

**Published:** 2015-03-12

**Authors:** Mahbub Alam, Paul L Voss

**Affiliations:** 1 Georgia Institute of Technology, School of Electrical and Computer Engineering, Atlanta, Georgia 30324-0250, USA; 2 UMI 2958 Georgia Tech-CNRS, Georgia Tech Lorraine, 2–3 Rue Marconi, 57070 Metz, France

**Keywords:** decoherence, graphene nanoribbon, phase coherence, photodetector, quantum interference, resonant tunneling

## Abstract

In this work, a graphene quantum interference (QI) photodetector was simulated in two regimes of operation. The structure consists of a graphene nanoribbon, Mach–Zehnder interferometer (MZI), which exhibits a strongly resonant transmission of electrons of specific energies. In the first regime of operation (that of a linear photodetector), low intensity light couples two resonant energy levels, resulting in scattering and differential transmission of current with an external quantum efficiency of up to 5.2%. In the second regime of operation, full current switching is caused by the phase decoherence of the current due to a strong photon flux in one or both of the interferometer arms in the same MZI structure. Graphene QI photodetectors have several distinct advantages: they are of very small size, they do not require p- and n-doped regions, and they exhibit a high external quantum efficiency.

## Introduction

Graphene, a single layer of carbon atoms arranged in a honeycomb lattice structure, has attracted much attention from researchers because of its exceptional electronic, mechanical and optical properties such as high electrical mobility, high thermal conductivity, high mechanical strength, linear energy dispersion around the Dirac point and strong light absorption from near-infrared to visible wavelengths [1–3]. Graphene also exhibits ballistic electron transport over unusually long lengths [4–8]. Researchers have recently measured a momentum relaxation length of 10 μm in graphene nano-ribbons at room temperature [4]. Up to this length, resistance is independent of length and Ohm’s law does not describe transport [9]. They have also demonstrated a phase-coherence length of 100 nm at room temperature, that is, up to this length the electrons keep their phase-coherent wave nature and interference phenomena can be observed [6,9]. With semiconductor device size approaching its limits, a potential path forward could be new device structures that use the wave property of electrons. One device structure that has attracted attention is the resonant tunneling diode, whose operation is based on quantum interference [10]. In graphene nanoribbons, a Mach–Zehnder interferometer (MZI) structure can be devised which gives the same transmittance pattern as that of a resonant tunneling diode for incoming electrons [11–14]. Photon-assisted tunneling through double quantum walls by spatial Rabi oscillation has also been studied [15–16]. In this paper we investigate the optoelectronic properties of this MZI structure formed by graphene nanoribbons and a possible application of this structure as photodetector. In a MZI structure, an electron in the ground, transverse mode goes through the device with a transmittance of one (*T* = 1) due to constructive interference at energies corresponding to longitudinal resonant modes. At these resonant energies, the electrons have a high density of states. In this paper we investigate for the first time the interaction of light in a graphene nanoribbon MZI structure and specifically we study the coupling of light between longitudinal resonant modes for both zigzag and armchair structures.

Graphene photodetectors have been studied in detail [2–3,17–19]. The primary distinguishing features of graphene photodetectors are: photodetection over a wide spectral range from infrared to ultraviolet wavelengths, a transit-time-limited bandwidth of approximately 1.5 THz and a high internal quantum efficiency of 15–30% [2–3,19]. The photocurrent generation mechanisms in graphene photodetectors include the photovoltaic effect, photothermoelectric effect, bolometric effect and phonon drag effect [3]. In the photovoltaic effect, the built-in electric field generated in the junction of p- and n-type graphene is utilized for separation of photogenerated electrons and holes. Photocurrent generation without a p–n junction and bias has also been demonstrated by utilizing the built-in electric field at the metal–graphene interface [20].

In this paper, we present the simulation results of two different approaches for an all-graphene (leads and device) nanoribbon photodetector with applied bias in a MZI structure. In the first part, we analyze the efficiency of the coupling of light between two resonant peaks of the MZI structure in a graphene nanoribbon. Each absorbed photon produces an electron and all of the photogenerated electrons are collected at the leads. This occurs because we are considering an all-graphene (both lead and device) structure, the calculated lifetime of the electron from photoexcitation is greater than the calculated transit time of the electron through the device and the device length is less than the mean free path of the electron. Half of the electrons collected at the leads contribute to the net current, resulting in an internal quantum efficiency of 50%. With proper bias and a high-pass frequency filter, this structure could be used to detect time-varying optical input with subwavelength resolution. In the second part, we analyze the total current switching caused by the phase decoherence of electrons by placing a strong photon flux in one or both of the interferometer arms.

This structure has the advantages that it does not require a p–n junction, it can operate at subwavelength resolution, its dimensions are very small, and that the photodetector has a high internal quantum efficiency of 50% and external quantum efficiency of up to 5.2%. By varying the device dimensions or using different resonant peaks, this structure can be used to detect light of various photon energies.

## Device geometry

**Figure 1:**
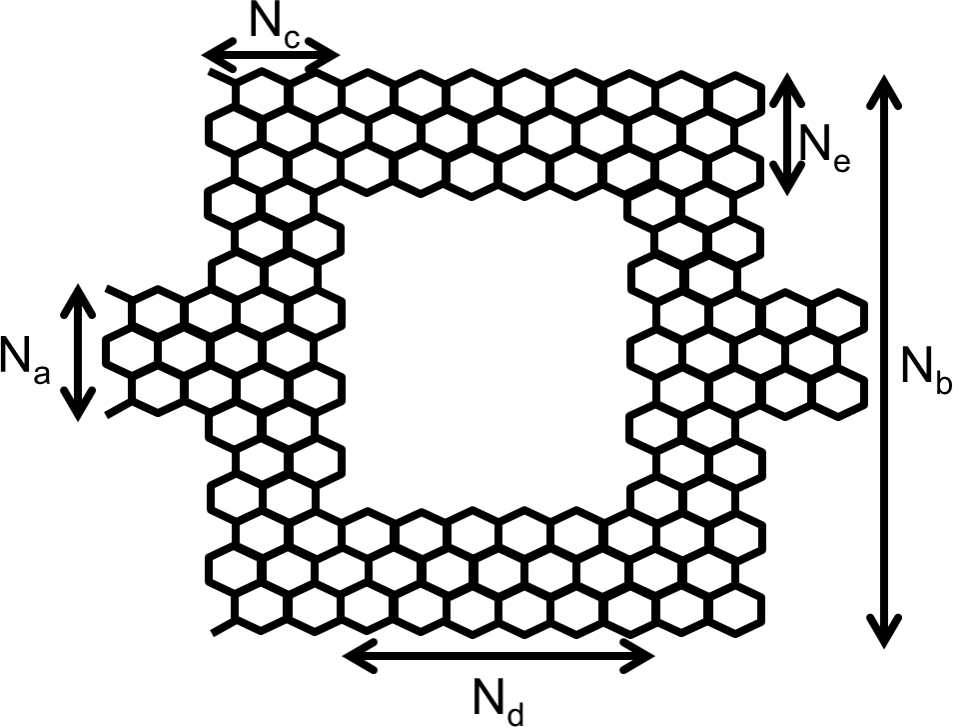
Graphene nanoribbon MZI structure (zigzag type).

**Figure 2:**
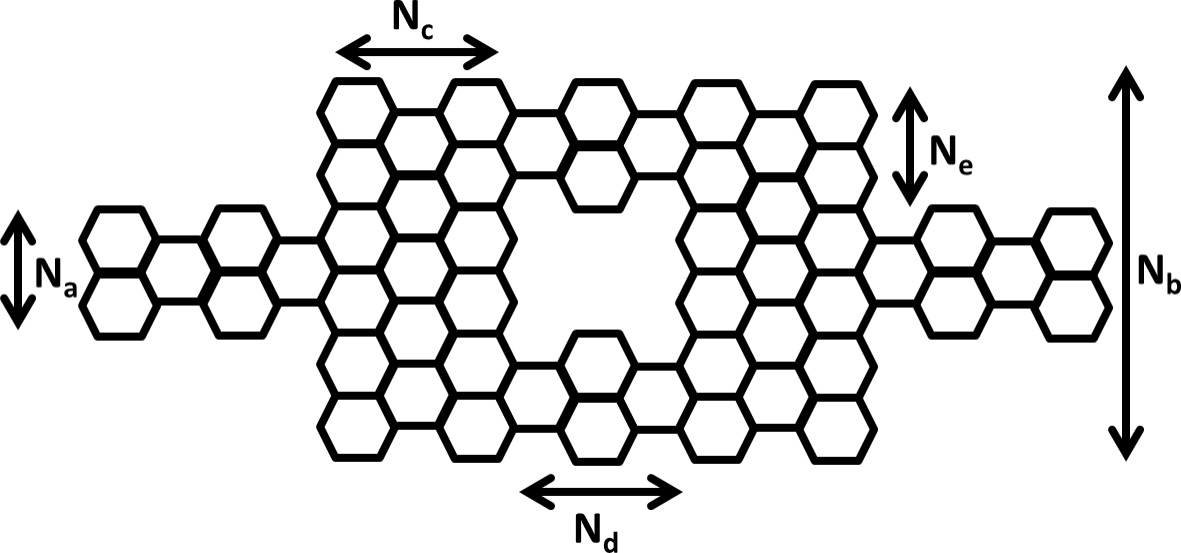
Graphene nanoribbon MZI structure (armchair type).

The device has a symmetric Mach–Zehnder-type interferometer structure as shown in Figure 1 and Figure 2. The device can be made of either armchair- or zigzag-type graphene nanoribbon.

This paper presents simulation results for both zigzag- and armchair-type nanoribbon structures. For the simulation of the zigzag-type we used *N_a_* = 1.136 nm (12 atoms), *N_b_* = 5.396 nm (52 atoms), *N_c_* = 0.986 nm (10 atoms), *N_d_* = 1.968 nm (18 atoms) and *N_e_* = 1.136 nm (12 atoms). For the armchair-type, *N_a_* = 0.738 nm (7 atoms), *N_b_* = 2.214 nm (19 atoms), *N_c_* = 0.71 nm (8 atoms), *N_d_* = 4.97 nm (48 atoms) and *N_e_* = 0.738 nm (7 atoms) were used. The lattice constant was set at 0.142 nm.

## Mathematical model

A non-equilibrium Green’s function (NEGF) formalism was used to calculate the current through the device [9,21–23]. Here, the Green’s function, *G^R^*, is the impulse response of the device and non-equilibrium implies that some voltage is applied for the current to flow. The Green’s function of the device, *G^R^*, is calculated from the Hamiltonian, *H_C_*, of the device and the self-energies, Σ*_l_*_1_, Σ*_l_*_2_ and Σ_photon_ (leads and photon) of the interaction. All calculations are performed in the energy domain and the position basis:

[1]
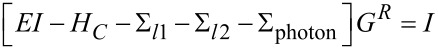


We have used a nearest neighbor, tight binding model to calculate the Hamiltonian, *H_C_*, of the device [11–13,24–29]. If the transfer energy, *t*, is greater than the energy range of interest, then the tight binding model (the discrete lattice representation) gives fairly accurate results [23,30]. In the second quantized form, the nearest neighbor, tight binding model has the following form:

[2]
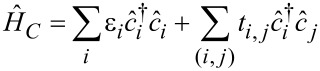


where ε*_i_* (= 0) is the on-site energy, *t_i,j_* = −*t* (*t* = 2.7 eV) is the transfer energy of the nearest neighbor sites and 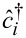 and 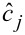 are the creation and annihilation operators of the π electron at sites *i* and *j*, respectively.

The electron correlation function, *G^n^*, and the hole correlation function, *G^p^*, (equivalent to density matrices) are calculated from the Green’s function of the device and the scattering functions Σ^in^ and Σ^out^ as

[3]
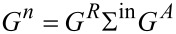


[4]
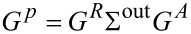


The scattering functions (Σ^in^ and Σ^out^) describe the rate at which electrons are scattered in and out for a certain energy level. This can be scattering into the device or out of the device at a certain energy (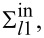 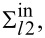 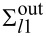 and 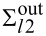) or scattering from one energy to another energy due to some interaction (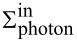 and 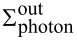). We assume a Fermi–Dirac distribution in the leads (*f*_1_ and *f*_2_). The Γ*_l_* functions are scattering rates provided that there are electrons and free states available and the Σ*_l_* functions are scattering rates which consider the availability of electrons and free states through Fermi–Dirac distribution and Pauli exclusion principle.

The scattering functions are calculated in the following way:

[5]
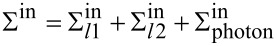


[6]
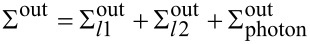


[7]
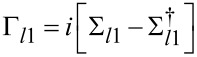


[8]
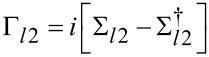


[9]
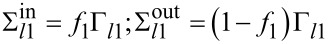


[10]
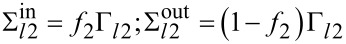


The transmittance, *T*, through the device can be calculated as

[11]
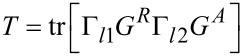


The effect of light illumination is incorporated in the calculation by the the inclusion of the Σ_photon_ term in the calculation of Green’s function as shown in Equation 1. The electron–photon interaction is calculated by the lowest order perturbation theory and self-consistent Born approximation [25,29,31–33]. The term lowest order implies that only single photon (linear) processes are included and the term self-consistent Born approximation implies that iteration is necessary until a self-consistent electron density in the ground and excited states is reached. The electron–photon interaction has the form *H*_elec−photon_ = (*e*/*m*_0_)*A*·*P*, where *A* is the vector potential and *P* is the momentum operator. If the vector potential, *A*, is expressed in the second quantized form, the electron–photon interaction in the position basis (after some manipulation) assumes the following form [31]:

[12]
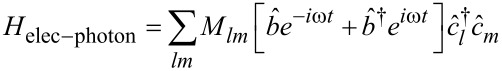


where

[13]
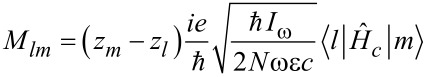


and

[14]
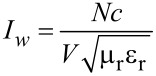


where *l* and *m* are site basis states. *z_m_* and *z_l_* are the positions of sites *m* and *l*, respectively. 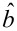 and 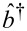 are the bosonic annihilation and creation operators, respectively. *I_w_* is the photon flux in units of photons/m^2^/s, *N* is the number of photons in a control volume of *V*, *c* is the speed of light, ε_r_ is the relative permittivity, μ_r_ is the relative permeability and ε is the absolute permittivity.

The photon scattering functions, 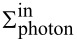 and 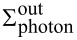, are calculated assuming monochromatic light and two energy levels for excitation.

[15]
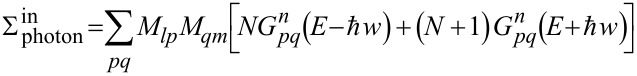


[16]
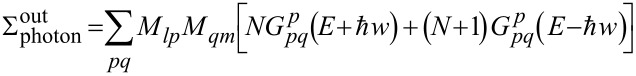


[17]
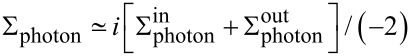


Both the acoustic phonon and optical phonon scattering have been neglected here because we are assuming phase coherent, ballistic transport and the mean free path for electrons is greater than the device length [25].

Knowing the electron and hole density functions (*G^n^* and *G^p^*) and the rate at which electrons are scattered in and out of the device (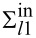 and 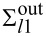), the energy resolved current (current per unit energy) is given by

[18]
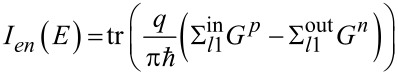


The total current is found by integrating the energy-resolved current over the energy range of the applied bias:

[19]
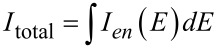


The total incoming scattering and outgoing scattering (Σ^in^ and Σ^out^) consists of incoming scattering from the leads and the photon (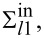 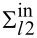 and 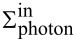) and outgoing scattering from the leads and the photon (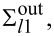 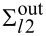 and 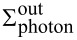) as shown in Equation 5 and Equation 6. If we want to calculate only the photoexcited portion of the electron and hole density matrices, then we consider the scattering due only to photons (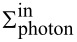 and 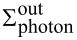) given by:

[20]
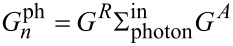


[21]
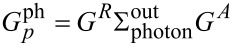


The energy-resolved photoexcited current is given by

[22]
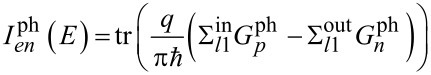


In this report, a Poisson solver was not used to account for the interaction of electrons present in the device. Since our applied voltage is quite low (0.1 eV) and there is no gate modulation in the device, the results obtained will still hold with good accuracy.

It should be mentioned here that we have used the tight binding model for both the armchair and zigzag structures. Zigzag edges of graphene nanoribbons have been shown to be magnetic [34–36]. Some reports used the tight binding model without magnetism in NEGF formalism for zigzag MZI structures [12–13] as well as other zigzag nanoribbon structures [37]. The device operation developed herein is not spin-dependent. We have not included the effect of magnetism in our tight binding Hamiltonian. However, an armchair nanoribbon does not have edge magnetism. Thus the tight binding Hamiltonian without magnetism can be used for an armchair nanoribbon without loss of accuracy. Our device operation is also valid for an armchair MZI structure although inclusion of the effect of magnetism is planned for our future studies of the zigzag MZI structure.

## Results and Discussion

**Figure 3:**
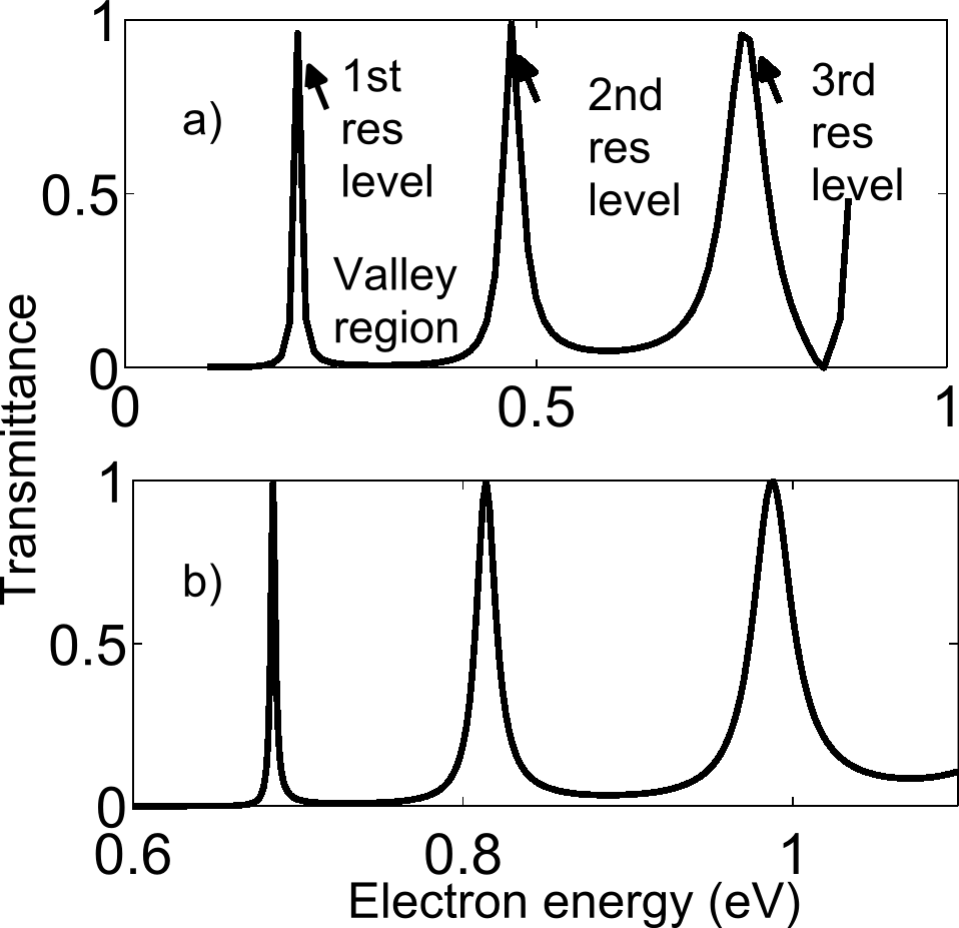
Transmittance versus change in electron energy for graphene nanoribbon MZI structure (a) zigzag type (b) armchair type.

The MZI structure in a graphene nanoribbon behaves like a resonant tunneling structure, meaning that at some energy, electrons pass through the structure as if there were no barriers. At this energy, the transmittance is one (*T = 1*) and constructive interference occurs. The energy at which this occurs is called the resonant energy level. There can be a 1st resonant level, a 2nd resonant level, etc. In contrast, at other energies, the electrons cannot pass through the device at all. At these energies the transmittance is zero (*T = 0*) and destructive interference occurs. These regions are called the valley regions. The modes described so far are the longitudinal resonant modes for the first transverse mode. At a higher energy and higher transverse modes, longitudinal resonant modes can also occur. The transmittance pattern for the zigzag structure is shown in Figure 3a and the transmittance pattern for armchair structure is shown in Figure 3b.

As the length of the middle arm, *N_d_*, increases, the longitudinal resonant peaks become sharper and the peaks become closer in energy. As the width of the nanoribbon, *N_a_*, increases, the higher transverse modes become closer in energy and the energy space available for longitudinal resonant modes to occur within a transverse mode decreases. Also, as the width *N_a_* increases, the longitudinal resonant peaks become sharper in energy. From our simulation results we see that by changing the device dimensions, we can detect photons of energy of 0.1 eV to 1 eV.

In the next section, we consider the response of the structure after light illumination. The two schemes for interaction with light are described below.

### Scheme 1: Coupling light between resonant peaks

**Figure 4:**
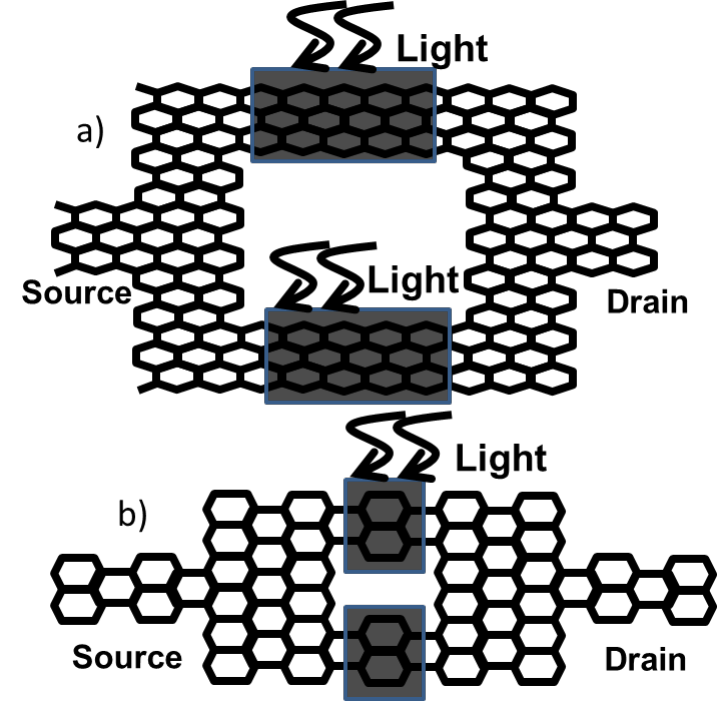
Device structure for light detection by coupling light between two resonant peaks. (top) zigzag structure (bottom) armchair structure.

By illuminating on both the interferometer arms as is shown in Figure 4, this structure can be operated as a photodetector. Upon illumination, electrons in the low-energy level (1st resonant level) absorb the light and get transferred to the high-energy level (2nd resonant level) and are emitted from the device without any other kind of interaction. The calculated lifetime of the electron from photoexcitation is greater than the calculated transit time of the electron through the device. Since we are assuming that the device length is less than the mean free path of the electron, we are neglecting all phonon interactions here. The photocurrent flows through the leads because one of the leads (drain) cannot supply the electrons to fill up the holes in the device (because the Fermi level in the drain is lower than the Fermi level in the source due to the applied bias). All of the photogenerated electrons are collected in the leads. Half of these electrons contribute to the net photocurrent, resulting in an internal quantum efficiency of 50% for the device.

**Figure 5:**
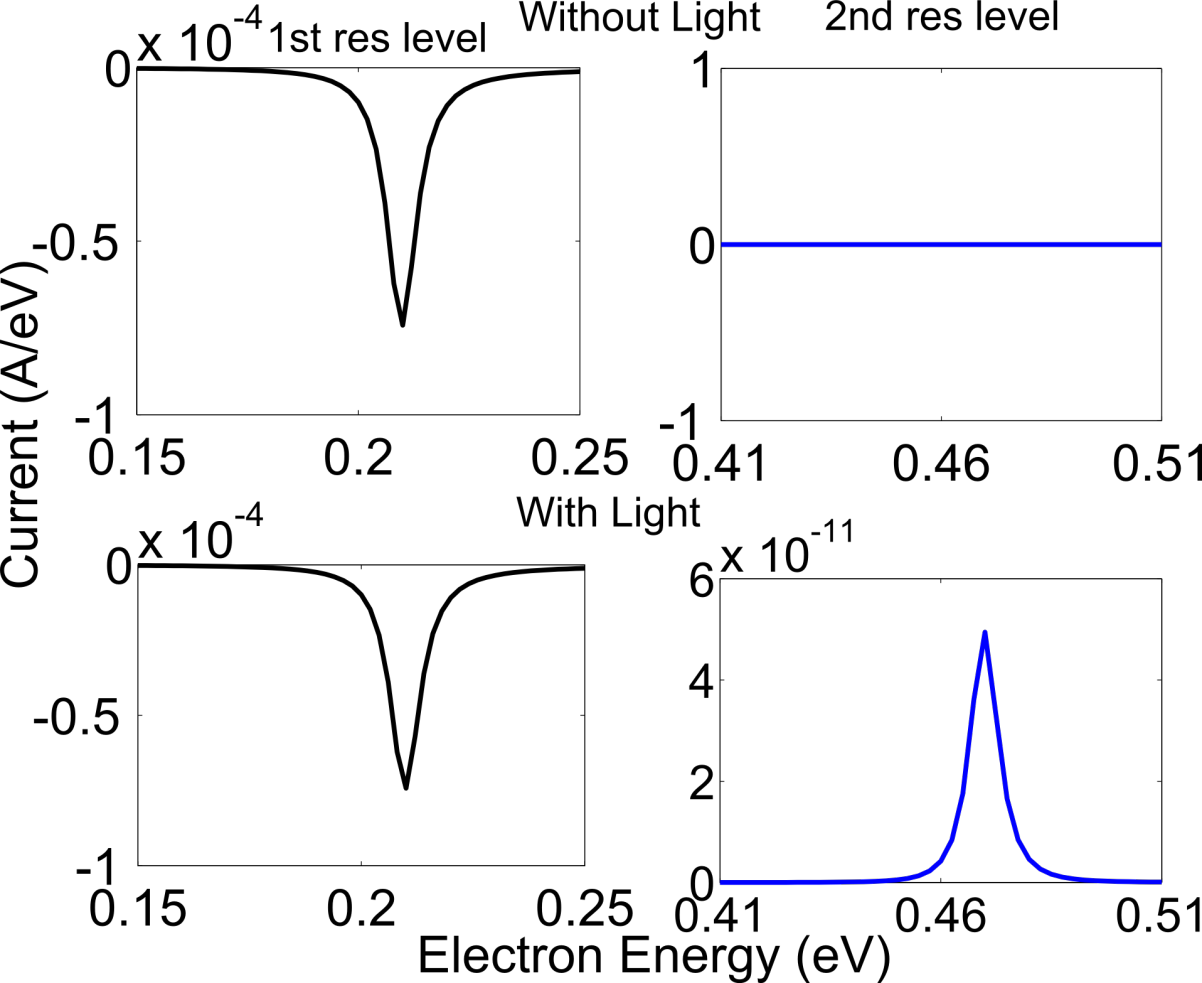
(zigzag structure) Current density versus electron energy for light detection by coupling light between two resonant peaks (top) without light (bottom) with light.

**Figure 6:**
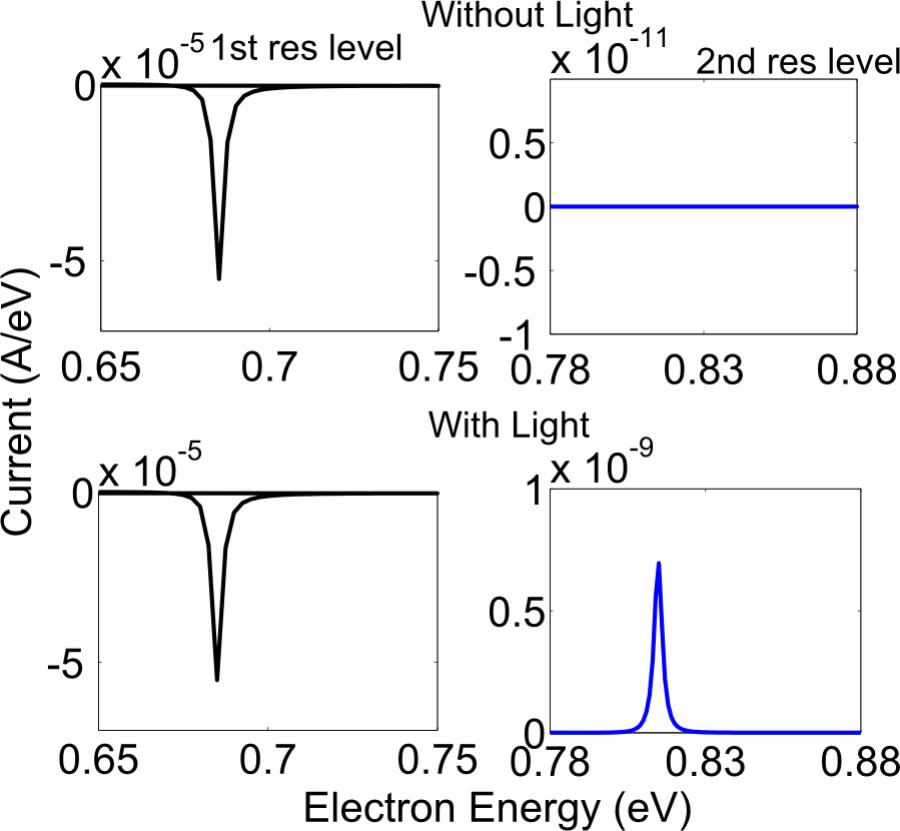
(armchair structure) Current density versus electron energy for light detection by coupling light between two resonant peaks (top) without light (bottom) with light.

The results of the interaction of light with the zigzag and armchair structures are shown in Figure 5 and Figure 6, respectively. For this simulation, the parameters used for the zigzag structure were an applied voltage of 0.1 eV, photon energy of 0.26 eV and a photon flux of 10^25^ photon/m^2^/s (4.16 × 10^6^ W/m^2^). The parameters used for armchair structure were an applied voltage of 0.1 eV, photon energy of 0.13 eV and a photon flux of 10^25^ photon/m^2^/s (2.08 × 10^6^ W/m^2^). For both the zigzag and armchair structures, the polarization of the applied electromagnetic field was along the length of the device. The full length of the middle, horizontal arms (216 (12 × 9 × 2) atoms for the zigzag structure and 336 (14 × 12 × 2) atoms for the armchair structure) was illuminated for this result. In the vertical arms, the absorption is two orders of magnitude less than the horizontal arms, so this result is equivalent to illuminating the entire structure. The voltage was applied in such a way that the first resonant level is within the applied voltage range. In top graphs of Figure 5 and Figure 6, we see that when there is no light, current flows in the low-energy level (1st resonant) but there is no current in the high-energy level (2nd resonant). Upon illumination, current flows in the high-energy level (2nd resonant) as shown in bottom part of Figure 5 and Figure 6. The currents shown in Figure 5 and Figure 6 are energy-resolved current, that is, the current per unit energy. A negative current indicates that electrons are entering the device.

**Figure 7:**
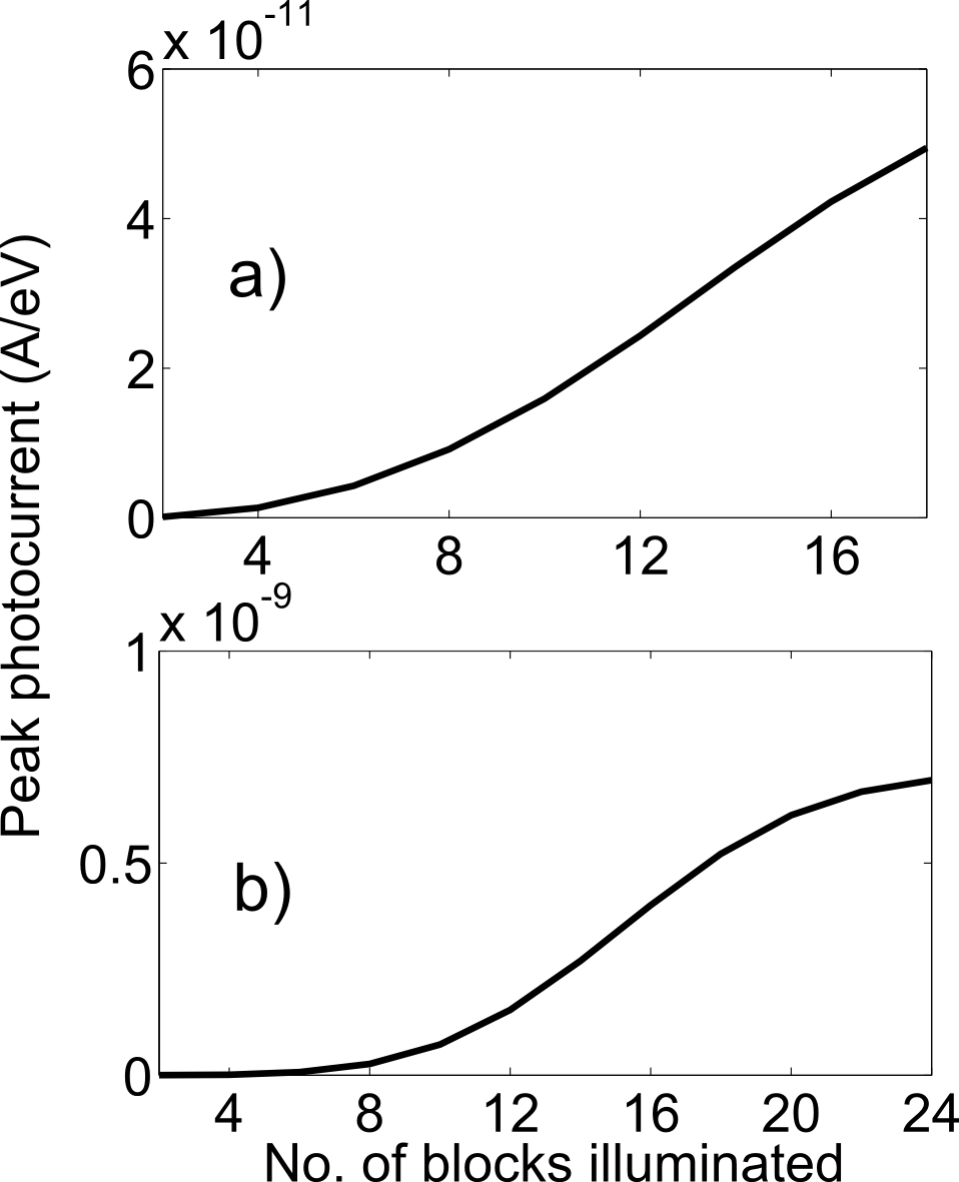
Variation of peak photocurrent with number of blocks illuminated. (a) zigzag structure, (b) armchair structure.

The photocurrent does not increase linearly with the number of atoms illuminated in the middle arm. The variation of the peak photocurrent with the number of blocks illuminated is shown in Figure 7. Each block contains 12 atoms in the zigzag structure and 14 atoms in the armchair structure. Initially, the peak photocurrent increases quadratically with the number of blocks illuminated and then the current saturates. This particular variation of current comes from the particular wave shape of the electron in position basis in the ground and excited states and Fermi’s golden rule, which is inherently contained in the NEGF formalism. The photocurrent is higher in the armchair structure compared with the zigzag structure. This is because in a zigzag structure, some neighboring atoms lie vertically and thus do not intercept the electric field because the polarization is in the horizontal direction. Also, the number of illuminated atoms (336) is greater in the armchair structure than for the number of illuminated atoms (216) in the zigzag structure.

It should be mentioned here that without excitation light, the bias current through the device is in the range of 10^−5^ A/eV and with light the photocurrent is in the range of 10^−11^ A/eV. Thus, some kind of differential measurement is needed to detect the current in the leads. Alternatively, a high-pass frequency filter can be used at the output of the device for the detection in the variation of light.

**Figure 8:**
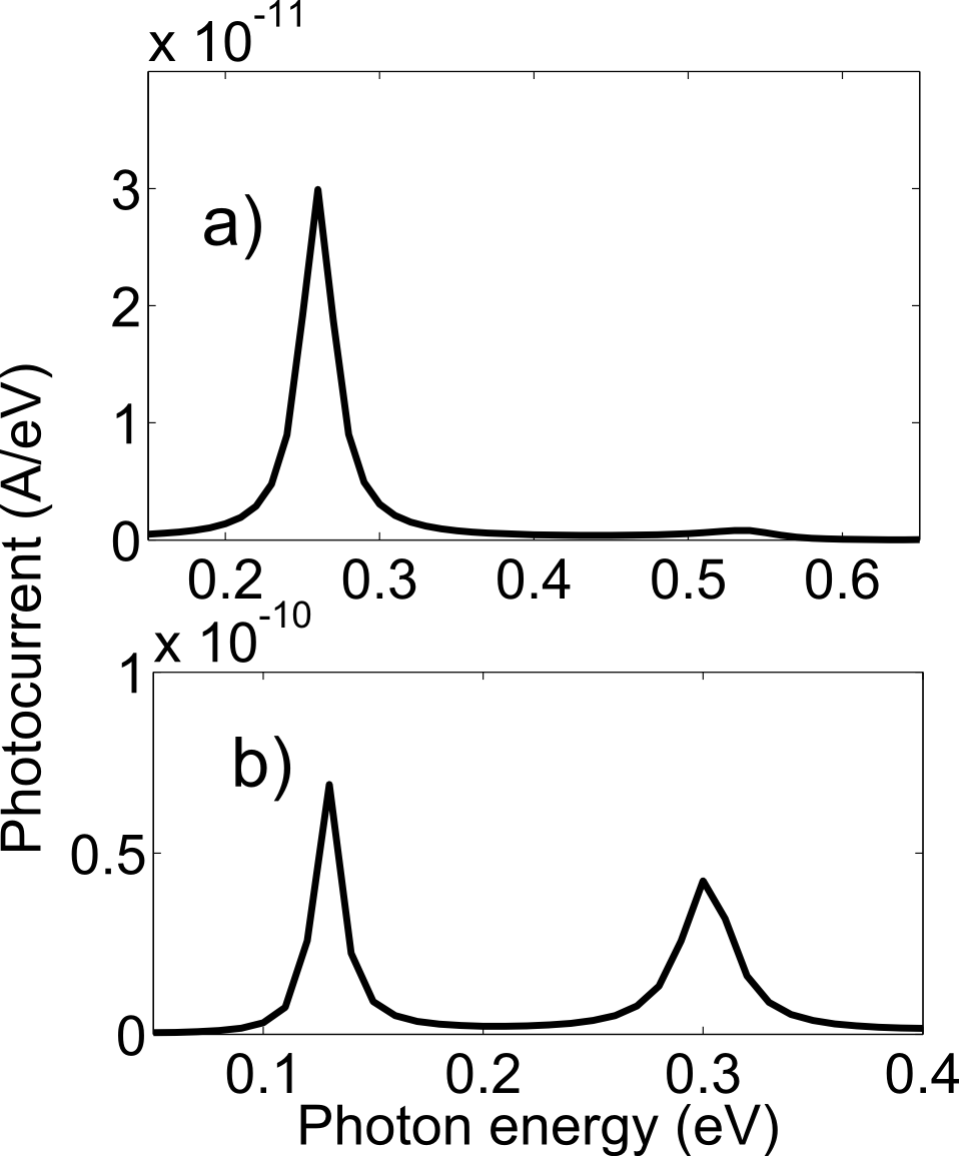
Variation of the peak photocurrent with photon energy. (a) Zigzag structure, (b) armchair structure.

With the appropriate bias, the device can also be used to detect the photon energy corresponding to the energy difference of any two resonant levels. The peak photocurrent variations with different photon energies are shown in Figure 8a and Figure 8b corresponding to zigzag and armchair structures, respectively. 144 (12 × 6 × 2) and 140 (14 × 5 × 2) atoms of zigzag and armchair structures, respectively, in the middle arm were illuminated for this result. 0.26 eV and 0.55 eV are the energy differences of 1st and 2nd resonant levels and 1st and 3rd resonant levels in the zigzag structure. 0.13 eV and 0.3 eV are the energy differences of 1st and 2nd resonant levels and 1st and 3rd resonant levels in the armchair structure.

If we integrate the energy-resolved photocurrent, we can calculate the total photocurrent through the device. For the integration, we have used Fermi–Dirac statistics at 300 K in the leads. Given the photocurrent, the external quantum efficiency of the device can be calculated as


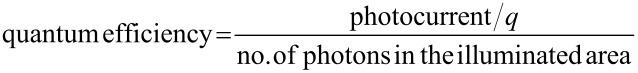


**Figure 9:**
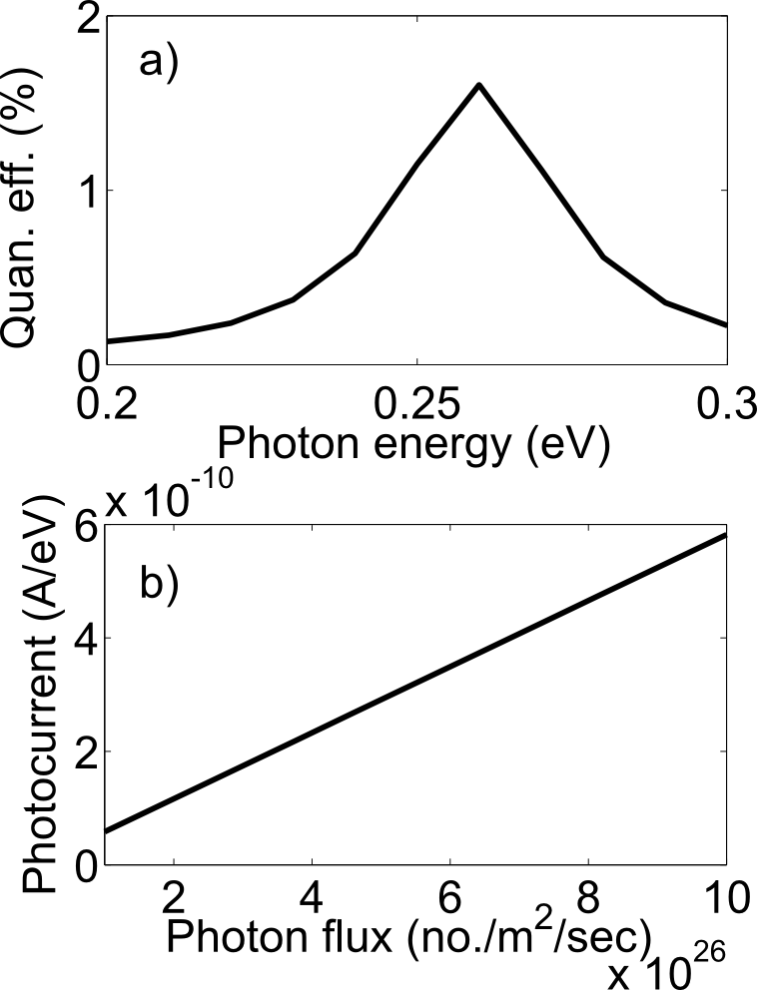
(a) The variation of the external quantum efficiency with photon energy. (b) Linear trend of peak photocurrent with photon flux (zigzag device).

**Figure 10:**
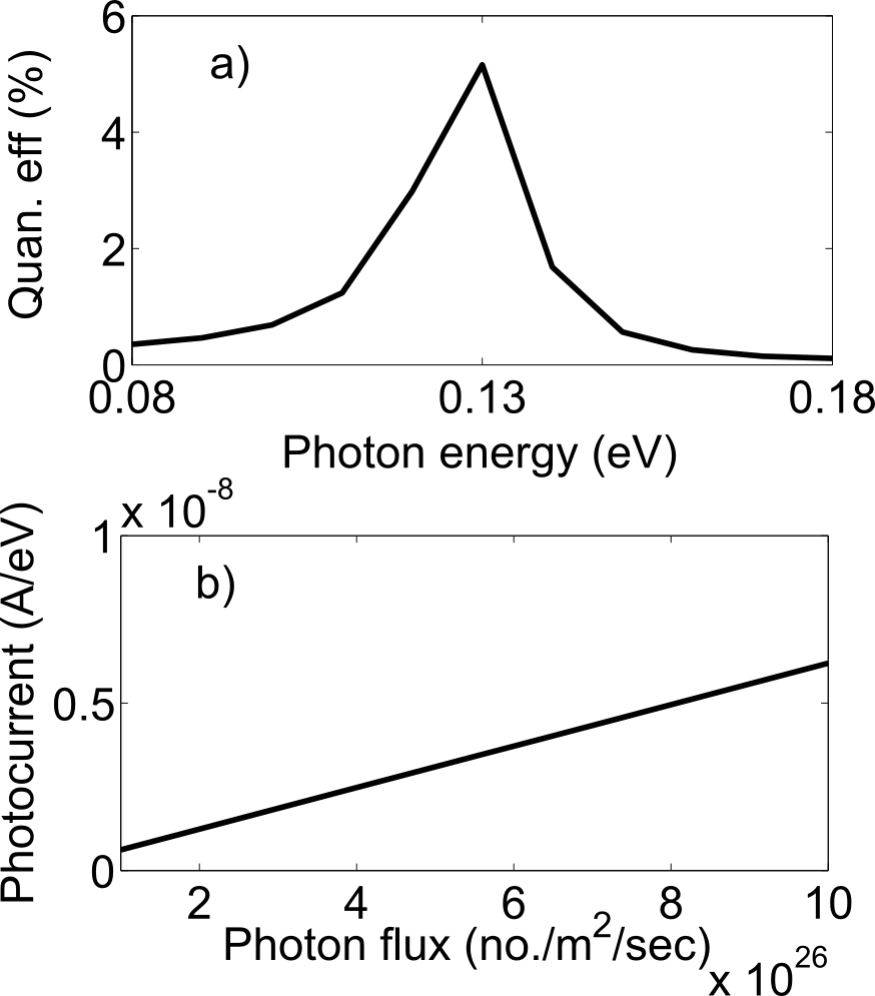
(a) Variation of the external quantum efficiency with photon energy. (b) Linear trend of the peak photocurrent with photon flux (armchair device).

For the zigzag structure, the external quantum efficiency reaches a maximum of 1.6% at a photon energy of 0.26 eV and for the armchair structure, the external quantum efficiency shows a peak of 5.2% at a photon energy of 0.13 eV, as shown in Figure 9a and Figure 10a. The photon energies 0.26 eV and 0.13 eV are the energy differences between the first two resonant levels in the zigzag and armchair structures, respectively. The quantum efficiencies are highest at 0.26 eV and 0.13 eV because the density of states is higher near the resonant energy levels. The external quantum efficiency remains constant with a photon flux of up to approximately 10^31^ photon/m^2^/s. The variation of the peak photocurrent with photon flux is shown in Figure 9b and Figure 10b.

We should mention here that the internal quantum efficiency for this device is 50%. In the literature, the reported value of experimentally determined internal quantum efficiency is 15–30% [19]. In the experimental result, the electron–hole pairs are separated by the built-in potential of the metal–semiconductor junction. The experimental internal quantum efficiency is lower because of electron–hole recombination from phonon scattering and scattering at the metal–semiconductor interface. The model presented herein does not allow for electron–hole recombination. The external quantum efficiency was 5.2% for the armchair structure and 1.6% for the zigzag structure. The photon absorption rate was 10.4% for the armchair structure and 3.2% for the zigzag structure. This is higher than the 2.3% absorption rate of bulk graphene due to two reasons. First, fully coherent transport of electrons occurs in the device, and second, the particular wave shapes of the electron in the 1st longitudinal resonant state and 2nd longitudinal resonant state within the first transverse mode in MZI structure contribute to the high absorption rate.

Experimentally, ballistic transport has been shown in graphene nanoribbons and MZI interferometer structures have been made in the graphene nanoribbons with a width of 40 nm [4]. The results presented here illustrate a MZI structure with a graphene nanoribbon width of 1 nm. The basic physics remains intact for devices of larger width and the device sizes will become smaller in future.

### Scheme 2: Decoherence with strong photon flux

#### One arm illuminated

In the previous section, it was shown that the photocurrent is quite low in comparison with the bias current through the device. In order to switch the total current a strong photon flux is needed. When the self energy (broadening) due to the incident light is comparable with the self energy (broadening) due to the contacts (i.e., when the lifetime of the electron from photoexcitation in the 1st and 2nd resonant levels is comparable with the transit time of the electron through the device), the incident light can cause phase decoherence for the majority of the propagating electrons. The lifetime of the electron, τ, is related to the self energy, Σ, by the following formula:

[23]
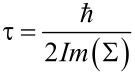


**Figure 11:**
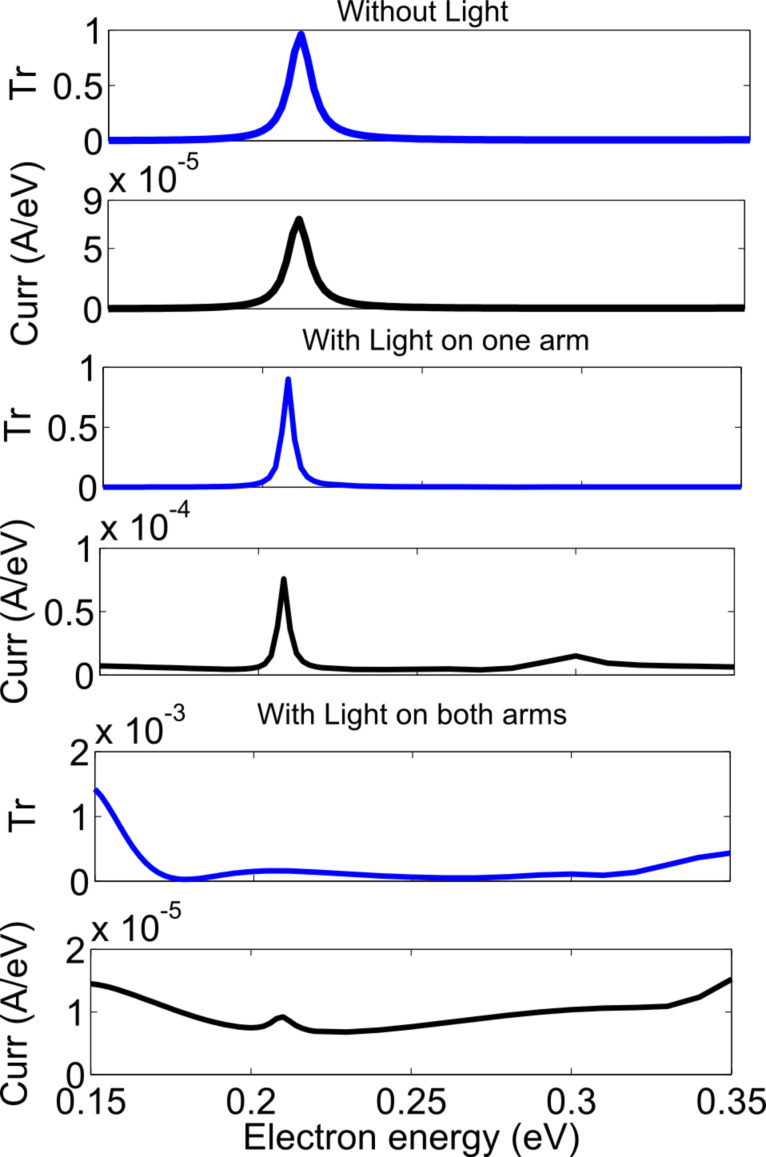
Transmittance and current density vs electron energy for strong photon flux (zigzag structure).

**Figure 12:**
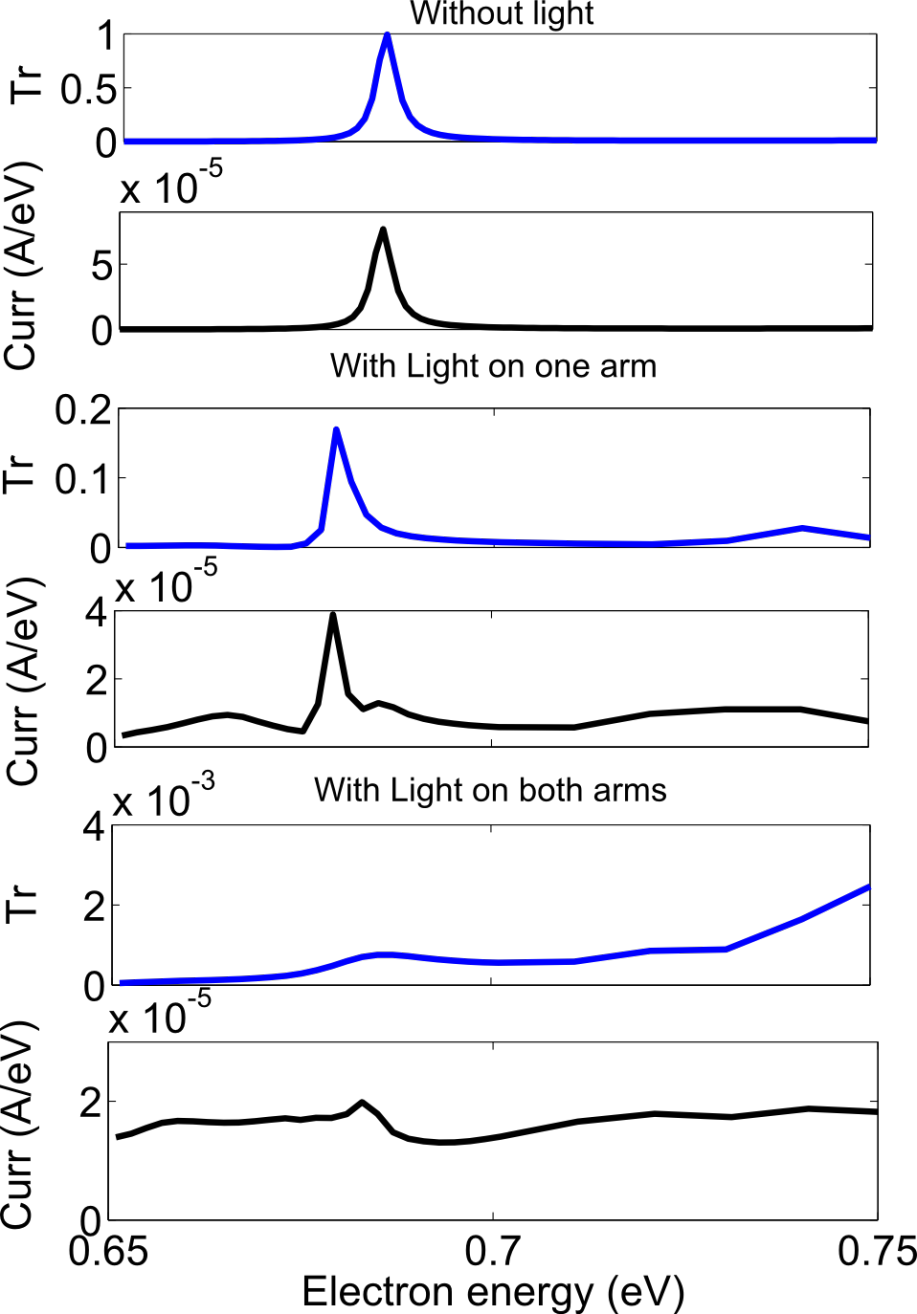
Transmittance and current density versus electron energy for a strong photon flux (armchair structure).

The results of the simulation with one of the MZI arms illuminated are shown in Figure 11 and Figure 12 and for zigzag and armchair structures, respectively. The parameters used for the zigzag structure were an applied voltage of 0.2 eV, a photon energy of 0.26 eV and a photon flux of 10^35^ photon/m^2^/s (4.16 × 10^16^ W/m^2^). The parameters for the armchair structures were an applied voltage of 0.1 eV, a photon energy of 0.13 eV and a photon flux of 10^35^ photon/m^2^/s (2.08 × 10^16^ W/m^2^). In Figure 11 and Figure 12, the simulation results for a large energy range are shown. For a practical device, the device will either be biased around the resonant level, where we want to reduce the current or around the valley region where we want to increase the current. With one arm illuminated, for the zigzag structure, as is shown in middle part of Figure 11, the transmittance in the peak region remains almost constant, but the peak position shifts by 0.002 eV, while the current in the peak region remains almost same, but the peak position shifts by 0.002 eV and the current in the valley region increases by 10 times. For the armchair structure, as is shown in middle part of Figure 12, the transmittance in the peak region decreases by 7 times and the peak position shifts by 0.006 eV, the current in the peak region remains almost same, but the peak position shifts by 0.006 eV and the current in the valley region increases by 10 times. With one arm illuminated, the coherent transmittance around the resonant level remains same for the zigzag structure but decreases by 7 times for the armchair structure. This is assumed to be because though the electrons lose their wave nature in one arm, yet resonant tunneling can still occur through the other arm. The destructive interference in the valley region is lifted due to the loss of coherent transport in one arm and thus incoherent current flows there.

#### Both arms illuminated

In order to reduce the current in the resonant energy level, both arms must be illuminated. When both arms are illuminated, scattering is induced in both arms, and the electrons lose their wave nature in both the arms, which effectively destroys the constructive interference. As stated before, the destructive interference in the valley region is also lifted. For the zigzag structure, as shown in the bottom part of Figure 11, the coherent transmittance in the peak region is reduced by a factor of 1000, the current in the peak region is reduced by a factor of 5, and the current in the valley region increases by a factor of 10 as compared to the values of these parameters (transmittance, peak current and valley current) without excitation light. For the armchair structure, as shown in the bottom part of Figure 12, the coherent transmittance in the peak region is reduced by a factor of 1000, the current in the peak region is reduced by a factor of 4, and the current in the valley region increases by a factor of 30 as compared to the values of these parameters (transmittance, peak current and valley current) without excitation light.

## Conclusion

We have proposed a graphene photodetector that makes use of quantum interference. We have shown that such a device can be operated as a linear photodetector that is most sensitive when the excitation light can couple two of the resonant energy levels in the graphene nanoribbon MZI structure. At this photon energy, the calculated external quantum efficiency was approximately 1.6% for the zigzag structure and 5.2% for the armchair structure. It is also possible to switch the total current in the device by causing a phase decoherence of electrons with a very strong photon flux. In this regime, the electrons lose their phase coherent, wave property and the ability to exhibit interference. This study is a step forward in analyzing the physics and potential performance of coherent electronic and optoelectronic devices.

## References

[1] Novoselov K S, Fal’ko V I, Colombo L, Gellert P R, Schwab M G, Kim K (2012). Nature.

[2] Xia F, Yan H, Avouris P (2013). Proc IEEE.

[3] Avouris P, Freitag M (2014). IEEE J Sel Top Quantum Electron.

[4] Baringhaus J, Ruan M, Edler F, Tejeda A, Sicot M, Taleb-Ibrahimi A, Li A-P, Jiang Z, Conrad E H, Berger C (2014). Nature.

[5] Miao F, Wijeratne S, Zhang Y, Coskun U C, Bao W, Lau C N (2007). Science.

[6] Berger C, Song Z, Li X, Wu X, Brown N, Naud C, Mayou D, Li T, Hass J, Marchenkov A N (2006). Science.

[7] Heersche H B, Jarillo-Herrero P, Oostinga J B, Vandersypen L M K, Morpurgo A F (2007). Eur Phys J Special Topics.

[8] Du X, Skachko I, Barker A, Andrei E Y (2008). Nat Immunol.

[9] Datta S (2012). Lessons from Nanoelectronics - A New Perspective on Transport.

[10] Britnell L, Gorbachev R V, Geim A K, Ponomarenko L A, Mishchenko A, Greenaway M T, Fromhold T M, Novoselov K S, Eaves L (2013). Nat Commun.

[11] Munárriz J, Domínguez-Adame F, Malyshev A V (2011). Nanotechnology.

[12] Wu Z, Zhang Z Z, Chang K, Peeters F M (2010). Nanotechnology.

[13] Zhang Z Z, Chang K, Chan K S (2008). Appl Phys Lett.

[14] Stafford C A, Cardamone D M, Mazumdar S (2007). Nanotechnology.

[15] Stoof T, Nazarov Y (1996). Phys Rev B.

[16] Stafford C, Wingreen N (1996). Phys Rev Lett.

[17] Bonaccorso F, Sun Z, Hasan T, Ferrari A C (2010). Nat Photonics.

[18] Urich A, Unterrainer K, Mueller T (2011). Nano Lett.

[19] Mueller T, Xia F, Avouris P (2010). Nat Photonics.

[20] Xia F, Mueller T, Lin Y-M, Valdes-Garcia A, Avouris P (2009). Nat Nanotechnol.

[21] Datta S (2005). Quantum Transport: Atom to Transistor.

[22] Datta S (2003). Electronic Transport in Mesoscopic Systems.

[23] Datta S (2000). Superlattices Microstruct.

[24] Stewart D, Léonard F (2004). Phys Rev Lett.

[25] Guo J, Alam M A, Yoon Y (2006). Appl Phys Lett.

[26] Wohlthat S, Reimers J R, Hush N S (2010). Phys Rev B.

[27] Castro Neto A H, Guinea F, Peres N M R, Novoselov K S, Geim A K (2009). Rev Mod Phys.

[28] Mohammadpour H, Asgari A (2011). Physica E.

[29] Ostovari F, Moravvej-Farshi M K (2014). Appl Surf Sci.

[30] Marconcini P, Cresti A, Triozon F, Fiori G, Biel B, Niquet Y-M, Macucci M, Roche S (2012). ACS Nano.

[31] Henrickson L E (2002). J Appl Phys.

[32] Guo J (2005). J Appl Phys.

[33] Gao Q, Guo J (2012). J Appl Phys.

[34] Son Y-W, Cohen M L, Louie S G (2006). Nature.

[35] Wang Z F, Liu F (2011). Appl Phys Lett.

[36] Tao C, Jiao L, Yazyev O V, Chen Y-C, Feng J, Zhang X, Capaz R B, Tour J M, Zettl A, Louie S G (2011). Nat Phys.

[37] Ahsan S, Masum Habib K M, Neupane M R, Lake R K (2013). J Appl Phys.

